# Viral lumbosacral radiculitis (Elsberg syndrome) in Denmark

**DOI:** 10.1007/s15010-023-02113-9

**Published:** 2023-11-02

**Authors:** Pelle Trier Petersen, Jacob Bodilsen, Micha Phill Grønholm Jepsen, Lykke Larsen, Merete Storgaard, Birgitte Rønde Hansen, Hans Rudolf Lüttichau, Jannik Helweg-Larsen, Lothar Wiese, Christian Østergaard Andersen, Henrik Nielsen, Christian Thomas Brandt, Danish Study Group of Infections of the Brain (DASGIB)

**Affiliations:** 1 Department of Pulmonary and Infectious Diseases, Nordsjællands Hospital, Dyrehavevej 29, 3400 Hillerød, Denmark; 2 Faculty of Health and Medical Sciences, University of Copenhagen, Copenhagen, Denmark; 3 Department of Infectious Diseases, Aalborg University Hospital, Aalborg, Denmark; 4 Department of Clinical Medicine, Aalborg University, Aalborg, Denmark; 5 Department of Infectious Diseases, Odense University Hospital, Odense, Denmark; 6 Department of Infectious Diseases, Aarhus University Hospital, Aarhus, Denmark; 7 Department of Infectious Diseases, Hvidovre Hospital, Hvidovre, Denmark; 8 Department of Infectious Diseases, Herlev Hospital, Herlev, Denmark; 9 Department of Infectious Diseases, Rigshospitalet, Copenhagen, Denmark; 10 Department of Medicine, Zealand University Hospital, Roskilde, Denmark; 11 Department of Clinical Microbiology, Hvidovre Hospital, Hvidovre, Denmark

**Keywords:** Viral meningitis, Aseptic meningitis, Myelitis, Urinary retention, Herpesviridae, Aciclovir

## Abstract

**Purpose:**

To describe clinical features and outcomes of viral lumbosacral radiculitis (Elsberg syndrome).

**Methods:**

Nationwide population-based cohort study of all adults hospitalised for viral lumbosacral radiculitis at departments of infectious diseases in Denmark from 2015 to 2020.

**Results:**

Twenty-eight patients with viral lumbosacral radiculitis were included (mean annual incidence: 1.2/1,000,000 adults). The median age was 35 years (IQR 27–43), and 22/28 (79%) were female. All patients had urinary retention, with 17/28 (61%) needing a catheter. On admission, at least one sign or symptom of meningitis (headache, neck stiffness, photophobia/hyperacusis) was present in 18/22 (82%). Concurrent genital herpetic lesions were present in 11/24 (46%). The median cerebrospinal fluid leukocyte count was 153 cells/µL (IQR 31–514). Magnetic resonance imaging showed radiculitis/myelitis in 5/19 (26%). The microbiological diagnosis was herpes simplex virus type 2 in 19/28 (68%), varicella-zoster virus in 2/28 (7%), and unidentified in 7/28 (25%). Aciclovir/valaciclovir was administered in 27/28 (96%). At 30 days after discharge, 3/27 (11%) had persistent urinary retention with need of catheter. At 180 days after discharge, moderate disabilities (Glasgow Outcome Scale score of 4) were observed in 5/25 (20%).

**Conclusions:**

Urinary retention resolved within weeks in most patients with viral lumbosacral radiculitis, but moderate disabilities according to the Glasgow Outcome Scale were common at the end of follow-up.

## Introduction

“*During the past five years in a large number of spinal operations under our observation, 84 of which were performed by one of us, and among which were many cases of diseases of the terminal spinal segments, we have met with 5 cases so alike in their histories, in their clinical findings, and in the morbid appearances on the operating table that we have been led to class them together, in the belief that we have here a definite clinical and pathological entity.*” [1]

In 1914, neurosurgeon C. A. Elsberg and colleagues described five younger adults (aged 37–45 years) with a syndrome of urinary retention, constipation, and radicular pain, paraesthesia, and paresis of the lower limbs of unknown but presumed infectious or toxic aetiology [1]. Neurotropic viruses, primarily herpes simplex virus type 2 (HSV-2), were later identified as a cause of lumbosacral radiculitis or myeloradiculitis, and hence the term Elsberg syndrome has been used for this condition [2]. However, as previous research has been limited to case reports and a single retrospective cohort study, the symptomatology and paraclinical characteristics of viral lumbosacral radiculitis require further systematic description to improve timely diagnosis and treatment [3–5]. In addition, the temporal restitution, including the duration of urinary retention, has not yet been sufficiently elucidated [3–5]. This study described clinical features and outcomes of all adults hospitalised for viral lumbosacral radiculitis at departments of infectious diseases in Denmark from 2015 until 2020.

## Methods

### Design, setting, and patients

This nationwide population-based observational cohort study used data from the Danish Study Group for Infections of the Brain (DASGIB) database. The DASGIB database has previously been described and holds data on prospectively included patients hospitalised for infections of the central nervous system (CNS) at the departments of infectious diseases in Denmark since 2015 [6]. In the present study, all adults (≥ 18 years) in the DASGIB database hospitalised for viral lumbosacral radiculitis between Jan. 1st. 2015 and Jan. 1st. 2020 were included. Viral lumbosacral radiculitis was defined as urinary retention with either (1) detected viral DNA or RNA in the cerebrospinal fluid (CSF) by polymerase chain reaction (PCR) or (2) CSF leukocyte count > 10 cells/µL and other microbiological evidence of infection with a neurotropic virus (e.g., positive intrathecal antibody index, serology, or PCR from other relevant patient samples) or 3) CSF leukocyte count > 10 cells/µL and viral lumbosacral radiculitis considered the most likely diagnosis given all available information. Patients with multiple hospitalisations for viral lumbosacral radiculitis within the study period were included with reference to their first hospitalisation. Patients with myelitis located cranial to the T9 segment [5] or with encephalitis [7] were not included.

### Variables

Data on clinical features were obtained from the DASGIB database, except for data on urinary retention, need of urinary catheter, constipation, lower limb radicular pain, lower limb paraesthesia, and lower limb paresis, which were obtained retrospectively by review of medical records. Immunosuppression was alcohol abuse, intravenous substance abuse, organ transplantation, cancer (except non-melanoma skin cancer), diabetes mellitus, asplenia, HIV infection, primary immunodeficiency, prednisolone > 7.5 mg/day, or other immunosuppressive therapy.

### Outcomes

In the DASGIB database, data on functional outcomes were categorised according to the Glasgow Outcome Scale (GOS [1. Death; 2. Vegetative state; 3. Severe disability; 4. Moderate disability; 5. Good recovery]) and assessed at discharge and at outpatient follow-up visits approximately 30 days, 90 days, and 180 days after discharge [6]. In case of missing values, the last observation was carried forward if patients had a good recovery (i.e., a GOS score of 5) at the most recent hospital contact. For the present study, data on additional sequelae (including persistent urinary retention) at 30 days after discharge were obtained retrospectively by review of medical records.

### Statistical methods

Median and interquartile range (IQR) were reported for quantitative variables. Counts and percentages were reported for categorical variables. Annual incidences of viral lumbosacral radiculitis were calculated using the entire adult Danish population each year as the denominator. Population data were obtained from Statistics Denmark [8]. Data were compared with the Mann–Whitney *U* test. *P*-values were two-sided and considered significant at < 0.05. All analyses were performed in SAS Enterprise Guide 7.1.

## Results

A total of 29 cases of viral lumbosacral radiculitis in 28 patients were identified in the DASGIB database. In the 5-year study period, the mean annual incidence of viral lumbosacral radiculitis was 1.2 episodes (range 0.6–2.0) per 1,000,000 adults.

The median age was 35 years (IQR 27–43), and 22 (79%) of 28 patients were female (Table 1). Immunosuppression was present in one (4%) of 28 patients. The median duration of symptoms before hospital admission was 5 days (range 1–30). The referral diagnosis was available for 22 patients and was meningitis in six (27%), other infectious diseases in five (23%), viral radiculitis or Elsberg syndrome in four (18%), cauda equina syndrome or equivalent signs and symptoms in three (14%), and miscellaneous in four (18%). On admission, 23 (82%) of 28 patients reported headache, six (23%) of 26 patients presented with neck stiffness, and 12 (55%) of 22 patients described photophobia and/or hyperacusis. At least one of these three signs and symptoms of meningitis was present in 18 (82%) of 22 patients. Eleven (39%) of 28 patients had concurrent genital herpetic lesions. According to the case definition, all 28 patients had urinary retention, and 17 (61%) needed a urinary catheter during hospitalisation. A median prodromal phase of 6 days (IQR 3–12) preceded urinary retention in 23 (82%) of 28 patients. Additional signs and symptoms of radiculitis were constipation in 20 (71%) of 28 patients, lower limb radicular pain in 13 (46%) of 28 patients (bilateral in five patients), lower limb paraesthesia in 10 (36%) of 28 patients, and lower limb paresis in two (7%) of 27 patients. At least one of these four additional signs and symptoms of radiculitis was present in 23 (85%) of 27 patients.

**Table 1:** Clinical features on admission in adults with viral lumbosacral radiculitis

Clinical features	Viral lumbosacral radiculitis
	N = 28
Age, years	35 (27–43)
Sex, female	22/28 (79)
Full-time occupation	23/28 (82)
Immunosuppression	1/28 (4)
Previous aseptic meningitis	1/28 (4)
Duration of symptoms before admission, days	5 (3–16)
Prodromal phase preceding urinary retention	23/28 (82)
Duration of prodromal phase, days	6 (3–12)
Urinary retention^a^	28/28 (100)
Urinary catheter needed	17/28 (61)
Constipation	20/28 (71)
Lower limb radicular pain	13/28 (46)
Lower limb paraesthesia	10/28 (36)
Lower limb paresis	2/27 (7)
Concurrent genital herpetic lesions	11/24 (46)
Headache	23/28 (82)
Neck stiffness	6/26 (23)
Photophobia/hyperacusis	12/22 (55)
History of fever	16/27 (59)
Temperature ≥ 38.0° Celsius	8/25 (32)
GCS score < 15^b^	1/27 (4)
Radiculitis/myelitis on MRI	5/19 (26)
B-leukocytes, cells × 10^9^/L	8.0 (7.0–8.8)
C-reactive protein > 10 mg/L	2/27 (7)
CSF leukocyte, cells/µL	153 (31–514)
CSF neutrophil percentage	1 (0–4)
CSF protein, g/L	0.70 (0.46–1.31)

Quantitative data are median (interquartile range) and categorical data are n/N (%)

^a^Required by case definition

^b^Glasgow Coma Scale score < 15 for < 24 h

Radicular signs and symptoms (i.e., urinary retention, urinary catheter needed, lower limb radicular pain, lower limb paresthesia, and lower limb paresis) and MRI findings were during hospitalisation

**Fig. 1:**
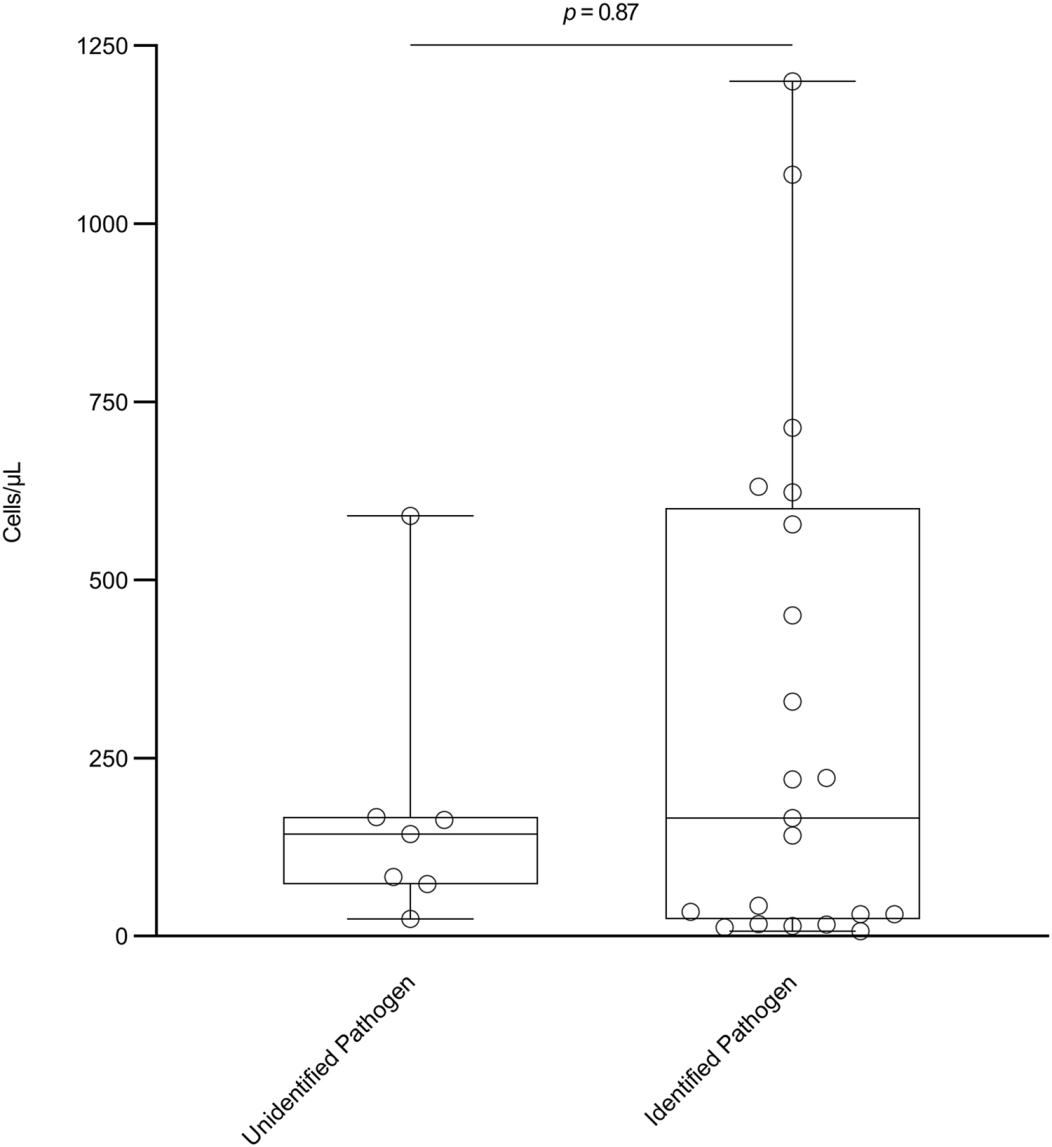
Cerebrospinal fluid leukocyte count by aetiology in adults with viral lumbosacral radiculitis. Box is interquartile range, horizontal line is median, and whiskers are range

**Fig. 2:**
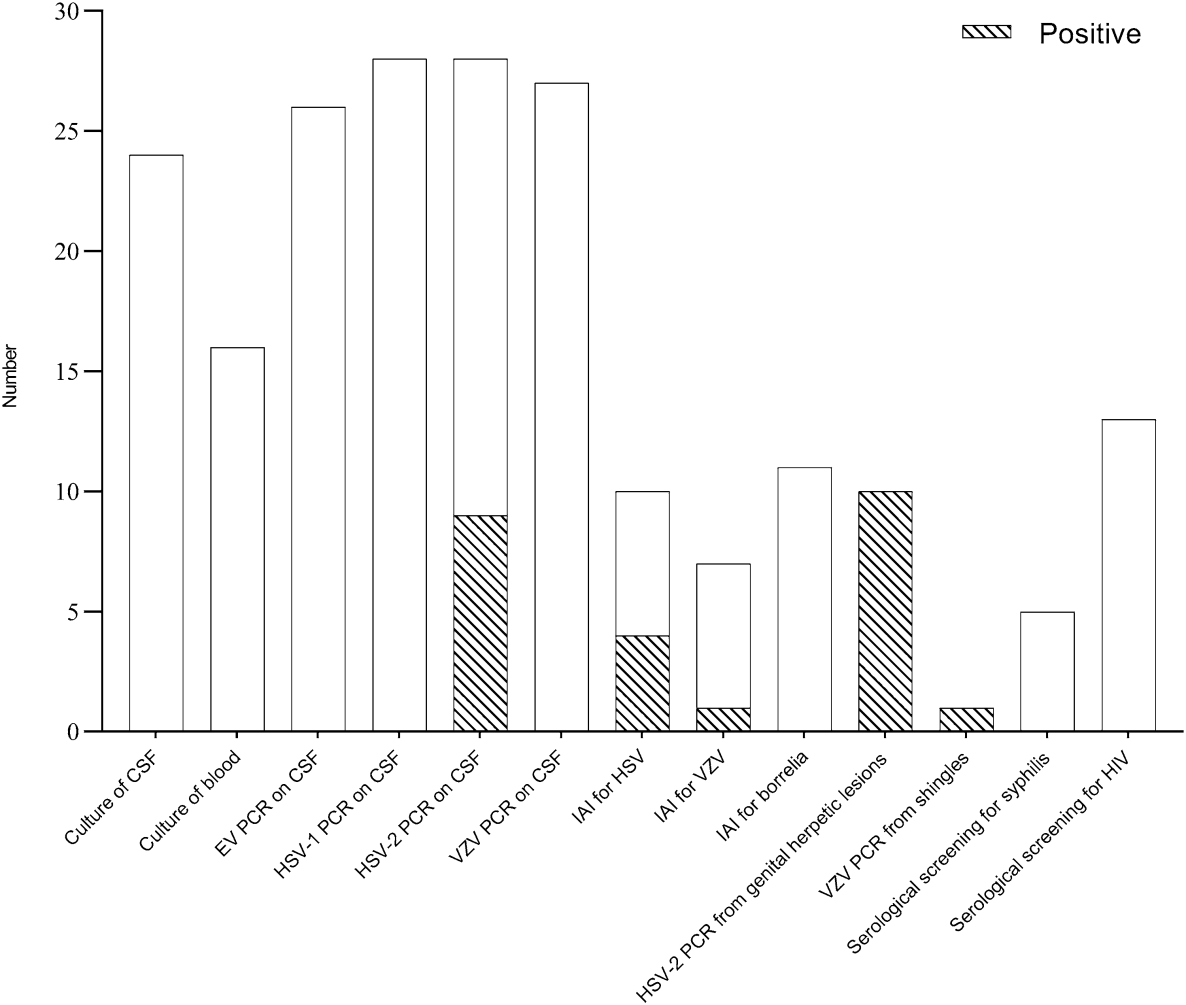
Microbiological tests in adults with viral lumbosacral radiculitis. *EV* enterovirus, *HSV* herpes simplex virus, *IAI* intrathecal antibody index

Magnetic resonance imaging (MRI) of the spinal cord was done in 19 (68%) of 28 patients, and five (26%) had findings suggestive of radiculitis and/or myelitis. Lumbar puncture was carried out in all patients at a median time of 6.0 h (IQR 2.2–21.7) from admission. The median CSF leukocyte count was 153 cells/µL (IQR 31–514) and was similar between patients with and without an identified aetiology (Fig. 1). The median CSF protein level was 0.70 g/L (IQR 0.46–1.31). The microbiological diagnosis was HSV-2 in 19 (68%) of 28 patients, varicella-zoster virus (VZV) in two (7%) of 28 patients, and remained unidentified in 7 (25%) of 28 patients. Among the 19 patients with HSV-2 lumbosacral radiculitis, the microbiological diagnosis was obtained by PCR on CSF in six (32%) patients, PCR from genital herpetic lesions in five (26%) patients, a combination of PCR on CSF and from genital herpetic lesions in four (21%) patients, intrathecal antibody index in three (16%) patients, and a combination of intrathecal antibody index and PCR from genital herpetic lesions in one (5%) patient (Fig. 2). Among the two patients with VZV lumbosacral radiculitis, the microbiological diagnosis was obtained by intrathecal antibody index and PCR from shingles. Among the seven patients without an identified aetiology, the microbiological work-up included PCR on CSF for herpes simplex virus type 1 (HSV-1), HSV-2, and enterovirus (n = 7), culture of CSF (n = 5), intrathecal antibody index for borrelia (n = 4), and herpes simplex virus and VZV (n = 2), and serological screening for HIV and syphilis (n = 3).

Twenty-seven (96%) of 28 patients were treated with intravenous acyclovir and/or valacyclovir for a median duration of 14 days (IQR 7–14). At least one dose of empiric antibiotics for acute bacterial meningitis was administered in 10 (36%) of 28 patients, and at least one dose of adjuvant dexamethasone was administered in four (14%) of 28 patients. The median duration of hospitalisation was 8 days (IQR 5–14). Four (14%) of 28 patients were readmitted within 30 days from discharge, and the cause of readmission was related to urinary dysfunction in three patients.

Moderate disabilities (GOS score of 4) were observed in 12 (43%) of 28 patients at discharge, 10 (36%) of 28 patients 30 days after discharge, seven (26%) of 27 patients 90 days after discharge, and five (20%) of 25 patients 180 days after discharge (Table 2). Data on additional sequelae at 30 days after discharge were available for 27 patients, and included persistent urinary retention with need of a urinary catheter in three (11%), headache in nine (33%), fatigue in five (19%), concentration difficulties in three (11%), lower limb dysesthesia in three (11%), lower limb radicular pain in two (7%), lower limb paraesthesia in two (7%), dysuria in two (7%), and lower limb paresis in one (4%; Table 3).

**Table 2:** Functional outcomes in adults with viral lumbosacral radiculitis

Glasgow outcome scale score^a^	Days after discharge			
	At discharge	30 days	90 days	180 days
	N = 28	N = 28^b^	N = 27^b^	N = 25^b^
4 (Moderate disability)	12 (43)	10 (36)	7 (26)	5 (20)
5 (Good recovery)	16 (57)	18 (64)	20 (74)	20 (80)

Data are presented as n (%)

^a^No patient had GOS scores of 1 (death), 2 (vegetative state) or 3 (severe disability)

^b^Glasgow Outcome Scale scores of 5 were carried forward for 2 (7%) of 28 patients at 30 days after discharge, 11 (41%) of 27 patients at 90 days after discharge, and 20 (80%) of 25 patients at 180 days after discharge

**Table 3:** Additional sequelae (including persistent urinary retention) at 30 days after discharge in adults with viral lumbosacral radiculitis

Sequelae	Viral lumbosacral radiculitis
	N = 27
Urinary retention with need of urinary catheter	3 (11)
Dysuria	2 (7)
Lower limb radicular pain	2 (7)
Lower limb dysesthesia	3 (11)
Lower limb paraesthesia	2 (7)
Lower limb paresis	1 (4)
Headache	9 (33)
Fatigue	5 (19)
Concentration difficulties	3 (11)

Data are presented as n (%)

## Discussion

This nationwide population-based cohort study described clinical features and outcomes of 28 patients with viral lumbosacral radiculitis, primarily caused by HSV-2. The patients were mostly younger adult females with acute-onset urinary retention, often with concurrent genital herpes on admission. Urinary retention resolved within weeks in most patients, but the functional outcome remained reduced in a substantial proportion at the end of follow-up 180 days after discharge.

HSV-2 was detected in 19 (68%) of 28 patients, which is consistent with the general perception that this pathogen is the most common cause of viral lumbosacral radiculitis. The neurotropism of HSV-2 is well-recognised, and lumbosacral radiculitis or myeloradiculitis is presumably a result of viral invasion of the lumbosacral nerve roots and lower spinal cord through axonal transport from either mucocutaneus surfaces in primary or nonprimary infection or dorsal root ganglia in reactivation [9, 10]. HSV-2 lumbosacral radiculitis may present with or without genital herpes [3, 4, 11], as in HSV-2 meningitis [12]. In the present study, concurrent genital herpetic lesions were present in 11 patients and were the site of sampling for the only positive microbiological tests in five patients. This observation emphasises the importance of a thorough and complete physical examination, including assessment of genital herpes in younger adults with urinary retention. Pain-related voiding difficulty is a primary differential diagnosis in patients with sacral herpes; however, in the present study, a diagnosis of viral lumbosacral radiculitis was supported by pleocytosis or positive viral PCR on CSF. Correspondingly, signs and symptoms of clinical meningitis (i.e., headache, neck stiffness, and photophobia and/or hyperacusis) were common on admission. Frequent coexistence of HSV-2 lumbosacral radiculitis and meningitis has also been reported in previous studies [13, 14]; thus, attention to radiculitis among patients with HSV-2 meningitis as well as meningitis among patients with viral lumbosacral radiculitis is recommended.

VZV was the likely cause of lumbosacral radiculitis in two patients in this study, and has also previously been identified in patients with lumbosacral radiculitis [5]. Furthermore, myelitis, radiculitis, and ganglionitis in other segments of the nervous system are well-described manifestations of VZV [15, 16]. A few reports have also identified Epstein–Barr virus, cytomegalovirus, HIV, and HSV-1 in patients with lumbosacral radiculitis [17–20], but none of these viruses was detected in the present study. In addition, some flaviviruses as well as human T-lymphotropic virus type 1 (HTLV-1) may cause urinary retention [21, 22]; however, as most of these viruses are not endemic in Denmark, they were considered unlikely aetiologies in the present study. Identification of a viral pathogen in lumbosacral radiculitis may reduce unnecessary examinations, allow for occasional targeted antiviral treatment, and provide information on prognosis for patients and physicians. In a cohort study by Savoldi et al., only three of 30 patients with lumbosacral radiculitis had a confirmed viral aetiology, which may partly be explained by incomplete and delayed microbiological testing [5]. Still, despite an extensive diagnostic evaluation, including the exclusion of neuroborreliosis in most cases, the aetiology remained unidentified in seven of 28 patients in the present study. In addition to undetected pathogens, autoimmune causes, such as autoimmune glial fibrillary acidic protein astrocytopathy, are potential aetiologies [23].

Following the case definition, all patients in this study had urinary retention. Patients with a less pronounced symptomatology (i.e., only urinary hesitation, faecal incontinence, or constipation) could, however, also be considered to have viral lumbosacral radiculitis [5]. In the present study, a short prodromal phase preceded urinary retention in 82% of patients. Similarly, febrile illness before urinary dysfunction was reported by 12 (71%) of 17 patients with viral lumbosacral meningomyelitis in a case series by Oates and Greenhouse [3]. Although prodromal symptoms are not always present, their occurrence may indicate infectious aetiology rather than structural or vascular causes [4, 5]. Furthermore, most patients were admitted within 1 week after onset of symptoms in the present study, which aligns with the conclusion by Savoldi et al., that a more insidious onset of the disease suggests non-infectious aetiology [5]. In addition to urinary retention, other signs and symptoms of radiculitis (i.e., constipation, lower limb radicular pain, lower limb paraesthesia, and lower limb paresis) were common among patients in the present study; however, MRI of the spinal cord was normal in most cases. Although radiological findings supported the diagnosis in the majority of patients in the study by Savoldi et al., no pathognomonic traits were identified [5]. Thus, viral lumbosacral radiculitis should not be excluded in the absence of radiological evidence and seems to be a clinical diagnosis supported by CSF findings in most cases.

In agreement with previous reports, urinary retention resolved within weeks in most patients in this study [3, 4]. However, the functional outcome assessed by the GOS was frequently reduced, and although it improved during extended follow-up, a substantial proportion of patients had not achieved full recovery at 180 days after discharge. All patients without full recovery had moderate disabilities on the GOS, which meant that they were unable to return to premorbid occupational or social activities [24]. Besides urinary dysfunction and other radicular complaints, sequelae were comparable with those reported by patients with HSV-2 meningitis [12]. Antiviral treatment with aciclovir or valaciclovir is generally recommended in viral lumbosacral radiculitis, though its effectiveness is not clearly established [5, 25]. Almost all patients in the present study received antiviral treatment, and thus analyses of potential effects were not meaningful.

## Limitations

This study has limitations. Firstly, as only patients with viral lumbosacral radiculitis managed at departments of infectious diseases were included, those treated at other departments (e.g., neurology, urology, and gynaecology) would have been missed. This would have underestimated incidences and potentially biased information on clinical features and outcomes. Secondly, functional outcomes were assessed by the GOS, which may be too crude to adequately capture sequelae in patients with viral neuroinfections without cerebral parenchymatous involvement. Additional sequelae, including persistent urinary retention, were, however, thoroughly described. Finally, some data were obtained retrospectively by review of medical records, with an increased risk of missing data and information bias.

## Conclusions

Viral lumbosacral radiculitis is a rare condition but should be considered in younger adults with acute-onset urinary retention, especially if genital herpetic lesions and clinical meningitis is present on admission. HSV-2 was the most common pathogen, but despite extensive diagnostic evaluation, the aetiology remained unidentified in a substantial proportion of patients. Confirming previous observations, urinary retention was transient in most patients, but one-in-five failed to return to normal daily living at the end of follow-up 180 days after discharge.

## Data Availability

Data are only available with permission from the Danish health authorities.
